# Optic Neuritis in Chlorosis

**Published:** 1904-06-11

**Authors:** 


### Hospital Clinics.

### OPTIC NEURITIS IN CHLOROSIS.

The occasional occurrence of optic neuritis in chlorotic women, which was first described by Hirschberg in 1879, and to which in this country attention was directed by Gowers at or about the same date, has during the last few years attracted notice in an increasing degree. The problem is certainly one of great interest, and is by no means destitute of importance. For whilst the cases, at least to judge from the number recorded, are not numerous, they occur with sufficient frequency to indicate that optic neuritis must be regarded as a possible complication in any case of chlorosis, and though early diagnosis and prompt treatment usually succeed in averting any injury to the sight, neglect of these conditions has on more than one occasion been followed by such a measure of optic atrophy or retinal damage as to lead to considerable reduction of the visual acuity. J. Jameson Evans [1] has lately reported a series of these cases and states his conviction "that the condition is far from being rare." The experience on which this belief is based has presumably been acquired in ophthalmic practice, and it may be doubted, therefore, whether it adequately covers the entire field. Chlorotic women who become sensible of any visual defect are, of course, likely to consult an ophthalmic surgeon, who may thus easily gain a disproportionate view of the frequency with which optic neuritis occurs in association with chlorosis. Physicians report only occasional instances. For example, Byrom Bramwell,[2] in an analysis of 314 cases of chlorosis, mentions optic neuritis as present only "in a small proportion of cases," and Clifford Allbutt [3] describes it as "discovered occasionally." At the same time it must be admitted that many, perhaps the majority, of patients with chlorosis are not examined with the ophthalmoscope and thus, as optic neuritis does not necessarily produce any visual defect, it may well be the case that many instances are overlooked by the physician. The increasing extent in which recognition is given to the truth that the ophthalmoscope is not less necessary to the physician than to the ophthalmic surgeon may be expected to settle the statistical question on a definite basis. In reference to diagnosis there are two mistakes possible, and each, if made, will in turn mislead the prognosis. In the first place the discovery of double optic neuritis not unnaturally may give rise to the suspicion that some more serious condition than chlorosis is the cause of the neuritis. On the other hand, there is danger that the mere existence of a more or less pronounced anæmia may be too readily accepted as an explanation, and hence a favourable prognosis may be framed only to be refuted by later events. Thus in a case reported by Wardrop Griffith [4] the patient was anæmic and had extreme neuro-retinitis but no other cerebral symptoms, yet after some time she became delirious and died, and a tumour of the occipital lobe was discovered at the autopsy. In short, whilst it is beyond question that double optic neuritis may be a feature of chlorosis and may entirely disappear under treatment by rest and iron, the acceptance of chlorosis as an explanation of an existing optic neuritis should only be granted after the most careful measures have been taken to exclude all other possible explanations. Cerebral tumours, as in the case quoted above, occasionally give for some time little or no indication of their presence other than optic neuritis, and the presence of anæmia is no guarantee of the absence of a tumour. Again, young women are known, though the experience is not a common one, to suffer from a form of kidney disease which numbers among its early symptoms some failure of vision due to a neuro-retinitis, and here again anæmia may be a conspicuous feature of the case, whilst dropsies and other frequent signs of kidney disease are absent. A similar remark may be made about the optic neuritis which occasionally complicates secondary syphilis. As opposed to all this is the fact that even though the case prove to be one purely of optic neuritis in chlorosis there may exist symptoms, in addition to the neuritis, which suggest an intra-cranial lesion. Headache, for example, is no uncommon feature in chlorosis, and from many recorded instances there seems good reason to believe that, whatever be the explanation, headache of a very severe character often precedes and accompanies the optic neuritis of chlorotic women. Much the same may be said of vomiting, and it may be questioned whether some of the cases in which recovery from pain in the head, vomiting, and double optic neuritis has been reported have not had an explanation other than that of "localised meningitis" which has been commonly attached to them. All this goes to show that the diagnostic problem associated with the occurrence of optic neuritis in the presence of anæmia is a somewhat complex one, and demands, when it presents itself, very delicate handling. There are, in addition, further possible difficulties, to judge from a case recorded by Ernest Clarke and C. O. Hawthorne.[5] In this instance the patient, in addition to evidences of anæmia and optic neuritis, had an ocular paralysis (sixth nerve) and absence of the knee-jerks. These facts, taken together with a complaint of severe headache, obviously suggest in the strongest possible manner the existence of some gross intra-cranial lesion. Yet, after some weeks' rest in bed and the administration of iron in full doses, the patient made a complete recovery, except for some slight depreciation of the vision of one eye. It can hardly be an exaggeration to say that in such a case, and in others more or less closely allied to it, there cannot be an absolutely confident diagnosis until time for observation of the effects of treatment has been granted. Suppose, for example, in this instance the treatment of the anæmia had failed to modify the neuritis and other symptoms, the accuracy of the diagnosis would have come under suspicion and an intra-cranial lesion would have inevitably been regarded as the probable explanation. It is of the highest importance to recognise that the same principle applies to cases where potassium iodide has been ordered in the view that an existing optic neuritis is due to an intra-cranial tumour. Indeed, it may be said that the most immediate practical application of the observation that optic neuritis may be due to chlorosis is to rescue such patients from the diagnosis of intra-cranial tumour and from the consequent neglect of the one remedy, namely iron, on which their salvation depends. Even in the presence of other symptoms—delirium, coma, paralysis, for instance—which suggest intra-cranial disease, it should never be forgotten that in a young woman the subject of anæmia optic neuritis and the entire series of events may depend directly or indirectly on the chlorotic state. Equally it is necessary to remember that a double optic neuritis may for a considerable period be alone or almost alone as a clinical expression of an intra-cranial tumour, and that the presence of anæmia does not exclude such an explanation from the diagnosis. The clinical lesson seems to be that when a selection has been made, after a complete review of the case, between chlorosis on the one hand and tumour on the other as an explanation of an actual optic neuritis, the physician must be prepared to recast his opinions in accordance with the indications suggested by the results of the treatment adopted. To neglect potassium iodide in many cases of cerebral tumour is a therapeutic blunder of the first magnitude. The omission of iron from the scheme of treatment in chlorosis is hardly less serious. Hence when an optic neuritis is referred to one cause or the other, as the case may be, and the corresponding treatment fails to produce a favourable effect, it is high time to revise the diagnosis and to reconsider the entire position. It is incontestable that optic neuritis due to chlorosis has been met on more than one occasion by the measures properly directed against an intra-cranial tumour, with the result that the patient's power of vision has been permanently damaged. Only by a general recognition of the wisdom of ophthalmoscopic examination in all cases of anæmia, and of the fact that optic neuritis may be one of the results of such anæmia, can a similar disaster be prevented in the future.

The manner in which chlorosis leads to optic neuritis is very uncertain. Gowers[6] suggested that the neuritis is due to the abnormal quality of the blood in association with hypermetropia. C. O. Hawthorne[7] has argued in favour of the proposition that the immediate cause of the neuritis is a thrombosis of the intra-cranial veins or sinuses, whilst Jameson Evans, in the paper already quoted, advances degenerative changes in the vessel walls and changes in the composition and intra-vascular pressure of the blood with resulting discrepancy between intra-vascular and intra-ocular pressures as the most likely factors in the production of the neuritis. There is, therefore, much uncertainty on this aspect of the question, but it hardly affects the practical issues concerning diagnosis and treatment, at least so far as is at present known.

[1] Lancet, May 14, 1904. [2] Anæmia and Diseases of the Blood-forming Organs. [3] System of Medicine, vol. V. [4] Brit. Med. Jour., June 9, 1888. [5] Lancet, April 30, 1904. [6] Medical Opthalmoscopy. [7] Brit. Med. Jour., Feb. 8, 1902.

